# The Spring Distraction System for Growth-Friendly Surgical Treatment of Early Onset Scoliosis: A Preliminary Report on Clinical Results and Safety after Design Iterations in a Prospective Clinical Trial

**DOI:** 10.3390/jcm11133747

**Published:** 2022-06-28

**Authors:** Casper S. Tabeling, Justin V. C. Lemans, Anouk Top, E. Pauline Scholten, Hilde W. Stempels, Tom P. C. Schlösser, Keita Ito, René M. Castelein, Moyo C. Kruyt

**Affiliations:** 1 Department of Orthopaedic Surgery, University Medical Center Utrecht, 3584 CX Utrecht, The Netherlands; j.v.c.lemans-3@umcutrecht.nl (J.V.C.L.); a.top@students.uu.nl (A.T.); e.p.scholten-6@umcutrecht.nl (E.P.S.); h.w.stempels@umcutrecht.nl (H.W.S.); t.p.c.schlosser@umcutrecht.nl (T.P.C.S.); k.ito@tue.nl (K.I.); r.m.castelein@umcutrecht.nl (R.M.C.); 2 Orthopaedic Biomechanics, Department of Biomechanical Engineering, Eindhoven University of Technology, 5612 AZ Eindhoven, The Netherlands; 3 Department of Biomechanical Engineering, Twente University, 7522 NB Enschede, The Netherlands

**Keywords:** early onset scoliosis, dynamic, spring distraction system, growth-friendly, versatile, growth, curve correction, severe adverse events, unplanned returns to the operating room, patient-reported outcome measures

## Abstract

*Background*: The Spring Distraction System (SDS) is a dynamic growth-friendly implant to treat early onset scoliosis (EOS). Previous SDS studies showed promising results in terms of curve correction and complication profile. Nevertheless, complications did occur, which led to modifications in the implant design. The main iterations were a larger rod diameter and a more sagittal stable sliding mechanism. The purpose of this study was to investigate the performance of these iterations. *Methods*: All patients treated with the modified SDS and >1 year follow-up were included. Radiographic outcomes, severe adverse events (SAEs), unplanned returns to the operating room (UPRORs) and health-related quality of life (HRQoL) were investigated. *Results*: Seventeen EOS patients (three congenital, four idiopathic, nine neuromuscular, one syndromic) were included. Mean age at surgery was 9.5 ± 2.5 years. Similar to the first generation SDS, about 50% initial correction was achieved and maintained, and spinal growth was near physiological. Most importantly, SAEs and UPRORs were diminished and favorable with 0.10/patient/year. In addition, HRQoL increased during the first year postoperatively, indicating the implant was well accepted. *Conclusion*: These preliminary results indicate that the iterations of the SDS are effective in terms of reducing SAEs and UPRORs and increasing HRQoL in patients with EOS.

## 1. Introduction

Early onset scoliosis (EOS) is a three-dimensional (3D) curvature of the spine and trunk with mixed etiology, that occurs in children nine years of age or younger [1]. It is an uncommon condition with a complex group of underlying diagnoses, which, if left untreated, can cause progressive thoracic insufficiency and respiratory failure, ultimately leading to death [2,3]. The early onset nature of this disease puts children at a high risk of progression; therefore, early intervention is important [4]. The main goals in treating EOS are to control the 3D deformity of the thorax, allow for thoracic and pulmonary development, to minimize complications, procedures, hospitalizations and burden for the family, and to improve overall development of the child.

The initial treatment of EOS can be conservative, such as casting or bracing [5]. In syndromic or neuromuscular EOS, seating adaptations can also be helpful to increase comfort [6]. However, operative treatment is frequently required to allow adequate growth of the thorax [7,8,9]. This is important, because a T1–T12 height below 18 cm at skeletal maturity has been related to poor pulmonary function [10,11]. In order to facilitate spinal growth, growth-friendly strategies were developed. The traditional growing rods and vertical expandable prosthetic titanium rib devices require regular surgical distraction, leading to a burden on the patient, the family and the healthcare system, as well as concerns about mental development due to repeated anesthesia [12,13,14]. To overcome this huge disadvantage, magnetically controlled growing rods (MCGRs) were introduced, which allow distraction with an external magnet [15]. However, the MCGR has a high implant-related complication rate, which requires revision surgery in about 30–50% of patients after 2 years [16,17,18,19,20,21]. In addition, metal debris issues led to a temporary suspension of the CE certification and an international advice to limit MCGR implantations [22,23]. Although MCGR does not require surgical lengthening, the repeated out-patient clinic visits still pose a burden on patients and families [19,24]. Moreover, due to its rigid nature, the device is difficult to contour to the spine, especially in the sagittal plane, and because the spine is immobilized and unloaded, this may lead to implant failure, stress shielding and stiffening of the spine [25]. Finally, in repeated lengthenings, the “law of diminishing returns” is encountered, meaning that with every lengthening procedure, the yield of subsequent procedures tends to decrease [26].

**Figure 1:**
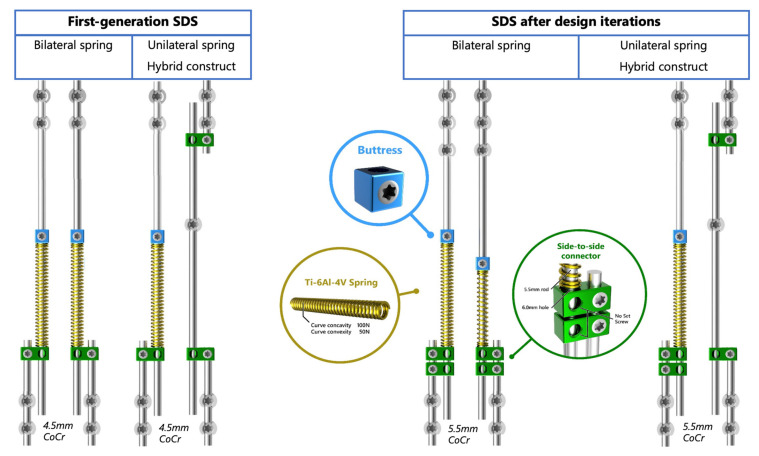
The Spring Distraction System. (**Left**) First-generation SDS with three components added to the 4.5 mm rods: a side-to-side connector (green) with one oversized hole that was kept unlocked, a compressed spring (gold) that provides a 75 N distraction force and a locking buttress (blue). (**Right**) Current SDS with 5.5 mm rods and an extra parallel connector. Moreover, an increased portfolio of springs with a 50 N and a 100 N version.

To counter these limitations, we developed a self-distracting dynamic implant, the Spring Distraction System (SDS) [27]. Its concept consists of compressed springs, mounted around conventional rods, which continuously distract the scoliotic spine. The first generation of SDS consisted of three main components that were added to the standard 4.5 mm growing rods: a side-to-side connector with one oversized hole, a compressed spring that provides a maximum 75 N distraction force at full compression and a locking buttress to pretension the spring over the rod (Figure 1).

Since the SDS is not yet registered as a medical device, all patients treated with the SDS are part of a prospective clinical trial. The results of the first 18 primary SDS patients at more than two years of follow-up were previously reported [28]. The main goals, to control the curve and maintain growth, were achieved. However, rod breakage and implant prominence due to increased kyphosis were a concern, leading to about 0.3 unplanned reoperations per patient per year [29]. To address these issues the design was improved by converting to 5.5 mm instead of 4.5 mm rods and adding one sliding connector to prevent kyphosis (Figure 1). Moreover, a 50 N and 100 N spring were added to the portfolio. This allowed more strategic positioning of the springs unilaterally as a concave spring, or bilaterally with symmetrical or asymmetrical springs.

The aim of this preliminary study was to assess the performance of the modified SDS with respect to curve maintenance, growth, severe adverse events (SAEs), rod breakage and increased kyphosis in particular, unplanned returns to the operating room (UPRORs) and patient reported outcomes, after a minimum follow-up of one year.

## 2. Materials and Methods

### 2.1. Study Design and Study Period

Data were collected from two prospective cohorts in which the SDS was implanted—the GRADS study and the BiPOWR study (ClinicalTrials.gov Identifier: NCT04021784)—between April 2019 and December 2020. The GRADS study is a single-center prospective cohort study investigating the SDS in all EOS patients. The BiPOWR study is a multicenter, randomized controlled clinical trial, comparing two growth-friendly distraction devices in nonambulant neuromuscular EOS patients indicated for bipolar fixation extended to the pelvis. Both studies were approved by the Institutional Review Board of the UMC Utrecht (METC 16/276; METC 18/179) and patients were included after informed consent. Inclusion criteria were all EOS patients that failed conservative treatment and were treated >1 year with an SDS that consisted of a 5.5 mm rod and double parallel connector. Exclusion criteria for SDS treatment were patients with connective tissue diseases that may not allow continuous distraction such as Marfan and Ehlers–Danlos syndrome, osteogenesis imperfecta and neurofibromatosis. This study followed the STROBE guideline for reporting observational studies (Supplementary Materials) [30].

### 2.2. Surgical Techniques and Implant Configurations

The surgical technique for placement of earlier versions of the SDS were described by Wijdicks et al. and Lemans et al. [27,28]. In short, small posterior incisions were used to create the proximal and distal anchors. Proximally, two or three consecutive pedicle screws were used per side and distally two pedicle screws or an iliosacral screw (Tanit^®^; Euros, SAS, La Ciotat, France) was placed. If the distraction device was applied unilaterally, usually a hybrid construct was made with a contralateral sliding rod fixed to the apex [31]. Somatosensory and motor-evoked potential monitoring were used intraoperatively. Skin-to-skin surgical time and blood loss were recorded.

For this study, we used standard 5.5 mm cobalt-chromium rods (CoCr) with 50 N, 75 N or 100 N medical-grade titanium (Ti6Al4V) spring(s). The spring was positioned on the sliding rod between a locking buttress (Stryker, Leesburg, VI, USA) and two oversized parallel connectors (NuVasive, San Diego, CA, USA) that allowed axial sliding without angulation (i.e., kyphosing within the connector). The spring behaved according to Hooke’s Law, therefore there was a decline in force with expansion. We mostly used the 100 N springs, which have a decline in force of 1.33 N/mm.

**Figure 2:**
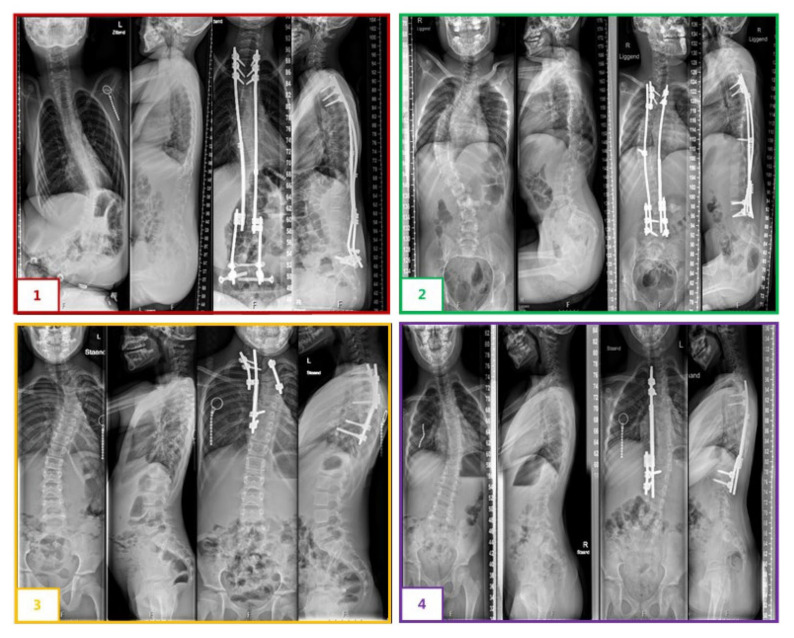
Different SDS configurations: (**1**) A 10-year-old male with neuromuscular scoliosis with a bilateral system with concave and convex springs fixated to S1, note the fully distracted spring after two years. (**2**) A 10-year-old male with an idiopathic-like scoliosis treated with a hybrid system with a concave spring and a convex sliding rod fixated with an apical screw. (**3**) A 7-year-old female with a congenital scoliosis treated with a unilateral system with a concave spring and convex hemi-epiphysiodesis. (**4**) A 9-year-old female with syndromic scoliosis treated with a unilateral system with a concave spring only.

We applied four different SDS configurations, depending on the curve magnitude and EOS etiology (Figure 2). Most idiopathic, congenital and syndromic patients received a hybrid configuration with an SDS on the concavity and a sliding rod with apical control on the convexity. Neuromuscular patients usually had a bilateral SDS configuration extending to the pelvis with a 100 N spring on the concavity and 50 N on the convexity. Postoperatively, all patients were allowed unrestricted physical activities.

### 2.3. Outcomes

Clinical data included sex, age at initial surgery and etiology of the scoliosis. Surgery time, blood loss and SAEs, categorized as implant-related (e.g., failure to distract) or procedure-related (e.g., surgical site infection), were scored. The number of UPRORs was separately scored. Health-related quality of life (HRQoL) was measured preoperatively, postoperatively and after one year with the validated Dutch EOSQ-24 questionnaire [32,33].

### 2.4. Radiographic Outcomes

All patients underwent full-spine erect coronal and sagittal radiographs preoperatively, postoperatively—as soon as the patient was fit for the radiograph—after one year and at the latest follow-up. Radiographic outcomes included Cobb angle magnitude of the primary (measured within the instrumented area) and secondary scoliotic curves, T1–T12 and T1–S1 height, and the T5–T12 kyphosis and L1–S1 lordosis were measured by two observers (CT, AT) in Surgimap Software v.2.3.2.1 (Nemaris Inc., New York, NY, USA). When the difference between observers was <5°, the mean of the two measurements was taken. Larger differences were discussed until a consensus was reached.

### 2.5. Statistical Analysis

Descriptive analyses were conducted in Microsoft Excel (Microsoft, Washington, IL, USA). Patient characteristics and outcome measures were reported as means with standard deviation or range. Graphs were created using GraphPad Prism Version 9.3.0 (GraphPad Software, San Diego, CA, USA).

## 3. Results

### 3.1. Patient Characteristics

In total, we included 17 patients (three congenital, four idiopathic, nine neuromuscular, one syndromic) with a mean age of 9.5 ± 2.5 years at surgery and a mean follow-up of 1.9 ± 0.5 years. One patient was lost to follow up at 11 months due to death, unrelated to the implant or surgical procedure. This patient suffered from spinal muscular dystrophy type 1 and died by sudden cardiac arrest due to hypoxia, caused by aspiration. All other patients were followed according to protocol. Mean surgery time was 169 min (range: 100–240) and mean blood loss was 395 mL (range: 100–700). Patients were discharged after a mean of 5 days (range: 4–9). Patient characteristics are summarized in Table 1.

### 3.2. Radiographic Outcomes

**Figure 3:**
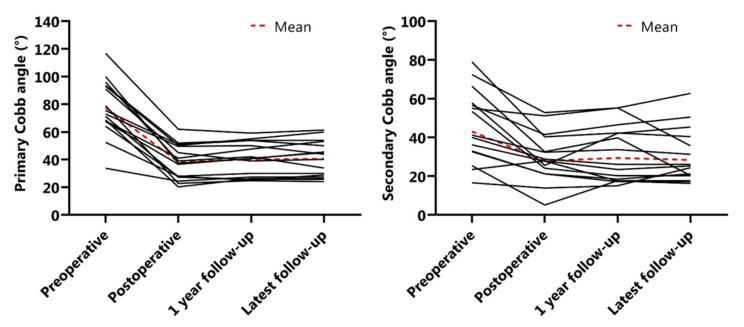
Coronal Cobb changes. (**Left**) Primary Cobb angle (°) changes over time. (**Right**) Secondary Cobb (°) angle over time.

**Figure 4:**
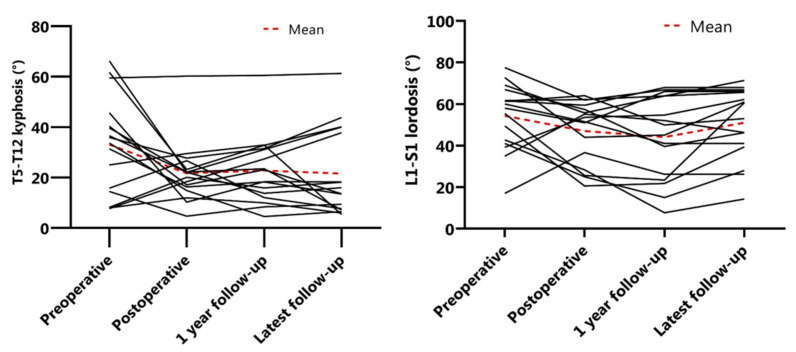
Sagittal Profiles. (**Left**) T5–T12 kyphosis (°) over time. (**Right**) L1–S1 lordosis (°) over time.

**Figure 5:**
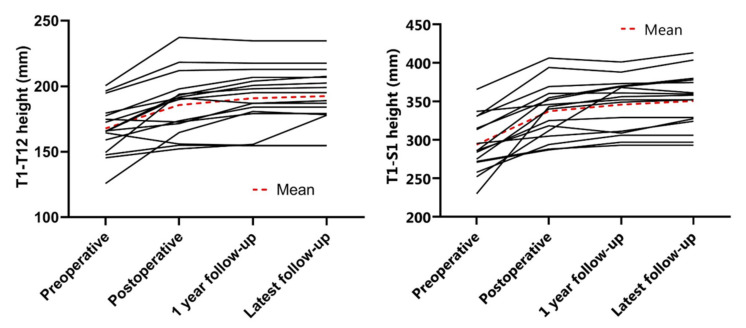
Spinal height changes. (**Left**) T1–T12 height (mm) over time. (**Right**) T1–S1 height (mm) over time.

The mean preoperative main Cobb angle was 78 ± 20°, which was reduced to 38 ± 12° (51% reduction) postoperatively. After one year of follow-up, the mean Cobb angle was 40 ± 12° and at latest follow-up 41 ± 13° (Figure 3). The secondary curve also reduced with surgery, from 43° to 21° and remained at 29° and 28° after one year and at latest follow-up, respectively (Figure 3). The mean preoperative T5–T12 kyphosis was 33 ± 19° and 22 ± 12° postoperatively, which was maintained at one year follow-up (Figure 4). Mean preoperative L1–S1 lordosis was 54 ± 16° and was 50 ± 18° at latest follow-up (Figure 4). Mean T1–T12 and T1–S1 height preoperatively to postoperatively was 167.8 ± 20.0 mm to 185.7 ± 24.1 mm and 293.8 ± 35.8 mm to 337.2 ± 35.8 mm, respectively (Figure 5). Mean T1–T12 and T1–S1 height gain due to surgery was 17.9 mm and 43.3 mm overall and growth in year one was 5.1 mm and 8.6 mm, respectively. All radiographic outcomes are summarized in Table 2.

### 3.3. Severe Adverse Events and Unplanned Returns to the Operating Room

An overview of SAEs and UPRORs is shown in Table 3. In two patients, progressive curves adjacent to the instrumented section were a reason for reoperation at 10 and 12 months. A third patient showed an unexpected high growth rate, which caused the spring to fully expand already after 11 months. This was not considered as an SAE, as it is a positive outcome of distraction, but it did require a reoperation 14 months after the initial surgery for a small spring retensioning. After initial surgery, this patient had a superficial surgical site infection, which was treated with oral antibiotics. Based on these three SAEs and three UPRORS during a follow-up of 1.9 years, we calculated 0.1 SAE and UPROR per patient per year. Most importantly, there were no implant related SAEs such as rod breakage or implant protrusion, as observed with the first-generation SDS.

### 3.4. Health-Related Quality of Life

**Figure 6:**
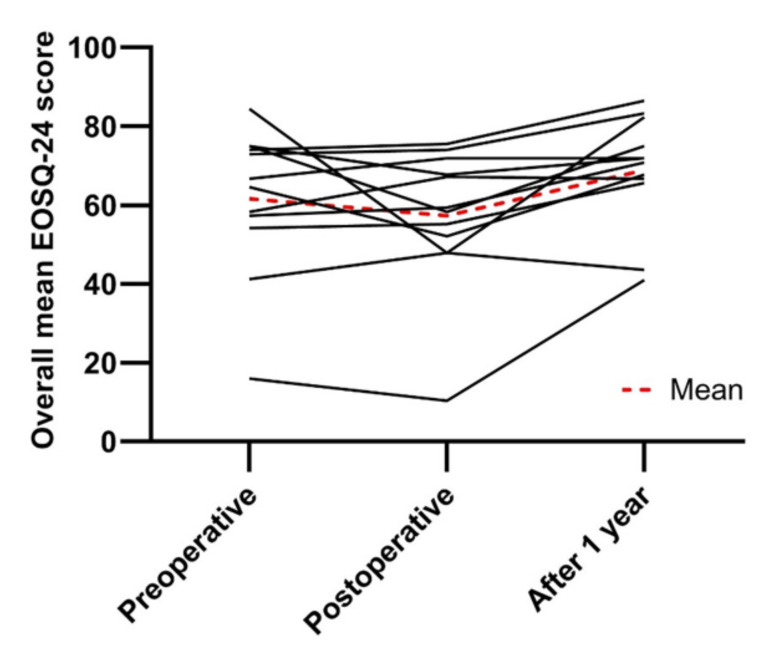
Overall mean scores of the Early-Onset Scoliosis Questionnaire plotted over time.

Twelve out of sixteen patients completed the EOSQ-24 questionnaires at all analyzed follow-up moments (Table 4). Mean overall scores initially decreased from 61.6 ± 18.5 preoperatively to 57.3 ± 17.7 postoperatively and thereafter improved to 68.9 ± 14.1 after one year of follow-up (Figure 6).

## 4. Discussion

In the current study we investigated an iteration of the SDS design, to mitigate material-related complications. In addition to good curve control and maintenance of growth, it appeared that the number of SAEs and especially UPRORs diminished compared to the first generation [28,29]. Obviously, as this is a preliminary study, the numbers were low, and a statistical analysis on an underpowered study was not worthwhile. However, the rate of SAEs and UPRORs decreased from about 0.3/patient/year, to <0.10/patient/year, which compared favorably to other growth-friendly systems [14,16,19,21,34,35]. Moreover, and maybe as a consequence, HRQoL improved, with even better scores one year after implantation compared to pre-implantation. This finding indicates that the implant may be better accepted than the first generation [29]. However, in the current study, fewer patients completed the EOSQ-24 at each follow-up, which could have caused a bias.

To prevent (excessive) kyphosis and subsequent material protrusion, we added an extra parallel connector in the current SDS design. This was effective, as the sagittal curves were well maintained after postoperative reduction. This differed from the previous SDS versions, where a substantial increase was observed that was intentional in selected cases, but a reason for revision in others. Since scoliosis in general can be considered as the result of a relative posterior shortening, we consider some increase of kyphosis beneficial, and future generations of SDS will be designed to accommodate that with rod contouring. Additionally, it should be mentioned that more rigidity in the sagittal plane may be a reason for rod failure over time, and although we have not seen this yet, it may appear in the coming years.

This preliminary study has obvious limitations, including the small patient cohort that was followed for a relatively short follow-up period. Furthermore, some patients were at the end of their growth spurt, which may explain less spinal growth after SDS treatment compared to the previous studies [27,29]. It is not possible to compare results of different SDS configurations, as the indication for uni- or bilateral springs is largely dependent on the etiology. Neuromuscular curves typically receive a bilateral SDS, in contrast to idiopathic curves. We will continue to further optimize the design, as metal wear between the sliding rod and connectors is still a concern, but also fundamental questions, such as which forces and configurations are optimal for specific conditions (e.g., etiology and curve type), demand for further investigations.

## 5. Conclusions

After a design iteration of the SDS, similar curve maintenance was observed compared to the previous system, with less implant-related complications and unplanned reoperations. These findings suggested that the earlier identified “room for improvement” indeed existed and allowed us to make an effective implant which may have less failures than the alternatively available systems.

## Figures and Tables

**Table 1:** Demographics.

Patient Characteristics	
Patients	17 (7 female)
Age at surgery (years)	9.5 ± 2.5
EOS etiology	
Congenital	3
Idiopathic	4
Neuromuscular	9
Syndromic	1
Surgery time skin-to-skin (minutes)	169 (range: 100–240) * (*N* = 17)
Blood loss (milliliter)	395 (range: 100–700) † (*N* = 17)
Time to discharge (days)	5 (range: 4–7)
Mean follow-up (years)	1.9 ± 0.5
Implant configuration (*N*)	
Concave + convex springs	9
Concave spring + convex apical screw	6
Concave spring + convex epiphysiodesis	1
Unilateral concave spring distraction only	1

* For one patient, surgery time was unavailable. † For one patient, blood loss was unavailable.

**Table 2:** Curve correction, sagittal profile and spinal growth.

	Preoperative	Postoperative	After 1 Year	Latest Follow-Up
Primary Cobb angle (°)	78 ± 20	38 ± 12	40 ± 12	41 ± 13
Secondary Cobb angle (°)	43 ± 21	28 ± 15	29 ± 16	28 ± 15
T5–T12 kyphosis (°)	33 ± 19	22 ± 12	23 ± 14	22 ± 17
L1–S1 lordosis (°)	54 ± 16	47 ± 15	44 ± 20	51 ± 17
T1–T12 height (mm)	167.8 ± 20.0	185.7 ± 24.1	190.8 ± 22.9	192.5 ± 21.5
T1–S1 height (mm)	293.8 ± 35.8	337.2 ± 35.8	345.8 ± 33.9	350.5 ± 35.6

**Table 3:** Overview of severe adverse events (SAEs) and unplanned returns to the operating room (UPRORs).

Patient	Sex	Age at SAE	Underlying Disease	Initial Surgery	SAEs	UPRORs and Treatment
P03	F	9.1 years	VACTERL association	SDS T2-L1 Bilateral hemivertebra resection and unilateral hemiepiphysiodesis T7-L1	Adding on above proximal anchor	Extension to C4
P06	F	11.2 years	Microcephalus	SDS T2-L4	Adding on below distal anchor	Extension to L5
P12	M	10.2 years	Myelomeningocele; Chiari II malformation	SDS T2-Ilium	SSI after initial surgery	Spring retension

**Table 4:** Health-related quality of life. Raw scores from 1–5 were transformed into scaled scores ranging between 0 and 100. Higher scores indicate better patient outcomes. Higher parental and financial burden scores indicate less negative impact in the past 4 weeks. The domain overall satisfaction is the mean of the child satisfaction and parental satisfaction domains. NB: 12/17 patients’ parents completed the questionnaire at each follow-up.

	Preoperative	Postoperative	After 1 Year
General health	67.7 ± 27.4	66.7 ± 17.9	70.8 ± 18.7
Pain/discomfort	60.4 ± 21.2	53.6 ± 19.7	61.5 ± 19.6
Pulmonary function	80.2 ± 25.8	78.1 ± 28.8	89.6 ± 13.9
Transfer	66.7 ± 30.8	57.3 ± 25.3	79.2 ± 23.4
Physical function	55.6 ± 31.8	46.5 ± 29.0	57.6 ± 31.5
Daily living	30.2 ± 24.1	30.2 ± 27.9	36.5 ± 25.3
Fatigue/energy level	67.7 ± 30.4	51.0 ± 27.4	66.7 ± 24.6
Emotion	62.5 ± 18.5	56.3 ± 18.8	74.0 ± 24.1
Parental burden	60.8 ± 26.2	60.0 ± 20.8	73.3 ± 19.0
Financial burden	83.3 ± 24.6	83.3 ± 30.8	93.8 ± 15.5
Overall satisfaction	62.5 ± 26.7	61.5 ± 17.2	70.8 ± 15.4
Overall mean score	61.6 ± 18.5	57.3 ± 17.7	68.9 ± 14.1

## Data Availability

Access to the data of this study is limited to the research team, study monitors and the data safety monitoring board. However, upon request, anonymized data can be made available by the principal investigator.

## Supplementary Materials

The following supporting information can be downloaded at: https://www.mdpi.com/article/10.3390/jcm11133747/s1, STROBE Statement—checklist of items that should be included in reports of observational studies.
